# Supplementary material for: Cross-Sectional Associations of Self-Reported Social/Emotional Support and Life Satisfaction with Smoking and Vaping Status in Adults
Source: Int J Environ Res Public Health. 2022 Aug 28;19(17):10722. doi: 10.3390/ijerph191710722 (PMC9517882; doi:10.3390/ijerph191710722)
Supplement: Supplementary file 1 [file ijerph-19-10722-s001.zip › ijerph-1838208-supplementary.pdf]

Table S1. The estimated adjusted odds ratios of smoking and vaping status for covariates.

Variables	Levels	Dual users	Ex-smokers	Exclusive smokers	Current vapers who were ex-smokers	Exclusive vapers
		(n=806)	(n=13,376)	(n=6,121)	(n=454)	(n=208)
Age						
	18-34	4.900 (2.750, 8.731)	0.328 (0.275, 0.390)	1.339 (1.065, 1.685)	2.605 (1.518, 4.470)	65.812 (8.604, 503.421)
	35-64	2.772 (1.704, 4.510)	0.591 (0.530, 0.658)	1.405 (1.182, 1.669)	2.126 (1.430, 3.161)	8.125 (1.142, 57.783)
	65+	Reference	Reference	Reference	Reference	Reference
Gender						
	Male	1.856 (1.409, 2.444)	1.751 (1.614, 1.898)	1.557 (1.389, 1.745)	1.920 (1.397, 2.638)	2.075 (1.105, 3.894)
	Female	Reference	Reference	Reference	Reference	Reference
Marital Status						
	Married	0.376 (0.220, 0.643)	0.621 (0.473, 0.816)	0.418 (0.318, 0.549)	0.395 (0.195, 0.799)	0.259 (0.079, 0.846)
	Divorced	0.667 (0.333, 1.337)	0.780 (0.584, 1.043)	1.113 (0.827, 1.499)	0.507 (0.235, 1.094)	0.810 (0.219, 2.994)
	Widowed	0.447 (0.213, 0.937)	0.695 (0.518, 0.932)	0.699)0.502, 0.972)	0.143 (0.053, 0.390)	2.233 (0.385, 12.951)
	Separated	0.694 (0.314, 1.531)	0.678 (0.423, 1.088)	1.053 (0.663, 1.671)	0.279 (0.064, 1.217)	<0.001
	Never Married	0.525 (0.306, 0.898)	0.364 (0.272, 0.486)	0.619 (0.470, 0.816)	0.304 (0.144, 0.641)	1.102 (0.437, 2.782)
	A member of an unmarried couple	Reference	Reference	Reference	Reference	Reference
Employment						
	Employed for wages	0.437 (0.288, 0.665)	0.741 (0.609, 0.902)	0.573 (0.461, 0.713)	1.038 (0.558, 1.930)	0.722 (0.141, 3.701)
	Self-employed	0.430 (0.236, 0.783)	0.759 (0.603, 0.955)	0.554 (0.420, 0.730)	0.622 (0.278, 1.392)	0.302 (0.047, 1.926)
	Out of work for 1 year or more	0.805 (0.409, 1.584)	0.901 (0.646, 1.257)	0.949 (0.647, 1.393)	0.702 (0.258, 1.911)	0.959 (0.104, 8.881)
	Out of work for less than 1 year	0.396 (0.187, 0.839)	0.759 (0.516, 1.117)	0.835 (0.582, 1.198)	1.558 (0.517, 4.695)	0.313 (0.048, 2.064)
	A homemaker	0.633 (0.291, 1.377)	0.722 (0.553, 0.944)	0.766 (0.557, 1.052)	1.024 (0.379, 2.770)	0.784 (0.056, 10.930)
	A student	0.199 (0.082, 0.484)	0.319 (0.200, 0.510)	0.148 (0.090, 0.245)	0.495 (0.173, 1.416)	1.441 (0.267, 7.774)
	Retired	0.378 (0.210, 0.681)	0.837 (0.683, 1.024)	0.533 (0.419, 0.679)	0.694 (0.377, 1.280)	0.938 (0.113, 7.759)
	Unable to work	Reference	Reference	Reference	Reference	Reference
General Health						
	Excellent	0.265 (0.138, 0.511)	0.550 (0.428, 0.706)	0.329 (0.246, 0.440)	0.163 (0.070, 0.377)	1.431 (0.257, 7.967)
	Very good	0.283 (0.163, 0.491)	0.636 (0.502, 0.807)	0.485 (0.374, 0.630)	0.377 (0.174, 0.816)	1.409 (0.263, 7.534)

Variables	Levels	Dual users (n=806)	Ex-smokers (n=13,376)	Exclusive smokers (n=6,121)	Current vapers who were ex-smokers (n=454)	Exclusive vapers (n=208)
	Good	0.592 (0.357, 0.980)	0.762 (0.602, 0.963)	0.704 (0.548, 0.905)	0.435 (0.203, 0.933)	0.919 (0.169, 4.989)
	Fair	0.454 (0.330, 0.899)	0.866 (0.678, 1.107)	0.956 (0.737, 1.239)	0.769 (0.355, 1.666)	1.487 (0.272, 8.134)
	Poor	Reference	Reference	Reference	Reference	Reference
Mental Health		1.034 (1.021, 1.047)	1.010 (1.003, 1.017)	1.028 (1.021, 1.035)	1.020 (1.000, 1.040)	1.020 (0.992, 1.049)

Table S2. Number of adult participants in each smoking/vaping category and age group.

Smoking/vaping status	Age: 18-34 (n = 7,459)	Age: 35-64 (n = 24,141)	Age: 65+ (n = 15,563)
**Dual users**	245	460	101
**Ex-smokers**	908	6,203	6,265
**Never users**	4,895	13,433	7,870
**Exclusive smokers**	1,103	3,755	1,263
**Current vapers who were ex-smokers**	126	266	62
**Exclusive vapers**	182	24	2
